# Introducing Mushroom Fruiting Patterns from the Swiss National Poisons Information Centre

**DOI:** 10.1371/journal.pone.0162314

**Published:** 2016-09-20

**Authors:** Katharina M. Schenk-Jäger, Simon Egli, David Hanimann, Beatrice Senn-Irlet, Hugo Kupferschmidt, Ulf Büntgen

**Affiliations:** 1 National Poisons Information Centre, Tox Info Suisse, Associated Institute of the University of Zurich, Zurich, Switzerland; 2 Swiss Federal Research Institute WSL, Birmensdorf, Switzerland; 3 Oeschger Centre for Climate Change Research, Bern, Switzerland; 4 Global Change Research Centre AS CR, Brno, Czech Republic; National University of Ireland - Galway, IRELAND

## Abstract

Changes in the ecology of macrofungi are poorly understood, not only because much of their life cycle is hidden belowground, but also because experiments often miss real-world complexity and most fruitbody inventories are limited in space and time. The National Poisons Information Centre ‘Tox Info Suisse’ provides countrywide 24hours/7days medical advice in case of poisonings since 1966. Here, we introduce a total of 12,126 mushroom-related phone calls that were received by Tox Info Suisse between 1966 and 2014. This indirect source of mycological information is dominated by the families of *Boletaceae* (11%), *Agaricaceae* (10%) and *Amanitaceae* (8%), which account for ~30% of all cases. Mushroom fruiting patterns revealed by the Poisons Centre inventory statistically resemble changes in fungal phenology, productivity and diversity as reflected by the Swiss National Data Centre ‘SwissFungi’. Although the newly developed Tox Info Suisse dataset provides an innovative basis for timely environmental research, caution is advised when interpreting some of the observed long-term changes and autumnal extremes. Uncertainty of the new record relates to possible data incompleteness, imprecise species description and/or identification, as well as the inclusion of cultivated and non-indigenous mushrooms. Nevertheless, we hope that the Tox Info Suisse inventory will stimulate and enable a variety of ecological-oriented follow-up studies.

## Introduction

Ecological responses to climatic fluctuations have recently been reported for different species from a wide range of taxa along various spatiotemporal scales around the world [1–4]. The observed reactions include, for instance, seasonal, year-to-year and longer term changes in the productivity, diversity and phenology of macrofungi [5–22]. A comprehensive review of climate variation effects on fungal fruiting has been recently provided [23].

Interannual fluctuations as well as decadal trends in the formation of fungal fruitbodies (mushroom productivity) and their composition (species diversity), as well as intra-annual shifts in the timing of their occurrence (seasonal phenology) have been related to a complex, multifaceted interplay of biotic and abiotic factors [23]. Possible drivers of fungal ecology may include among others, pathogens and pests, mycelial and metabolic activity, host physiology, resource competition, land-use/land-cover change, deforestation, increased harvesting, anthropogenic pollution, and climatic variation. In consequence, most of the observed spatial and temporal patterns in mushroom fruiting remain difficult to explain, because one-dimensional and causal explanations are rare. The general constrain in disentangling causes and consequences of long-term changes in fungal phenology, productivity and diversity mainly relates to unsystematic, incomplete, and/or insufficiently extended high-resolution mushroom inventories and monitoring programs [18]. Furthermore, experimental settings are generally too short and often also lack the necessary real-world complexity [23, 24].

At the same time, innovative approaches of (indirect) data mining and crowdsourcing have become instrumental in offering new insight into a wide range of systems and their underlying processes [25–30], for which (direct) evidence was not yet available. Citizen science projects [31–35], within and between different disciplines of environmental and life sciences, may indeed reveal novel and unexpected findings [36]. Posing the right questions to the right persons, applying the correct techniques and searching for allusive signals in hitherto unknown and putatively unsuitable archives [31], however, appears to be most critical and much more research is needed. In contrast to plants and animals, and despite their economic, social and ecological importance [37], fungal fruitbodies have been largely overlooked as objects for citizen science-based environmental studies [38].

According to the World Health Organization (WHO), poisons centres are specialized units advising on, or assisting with the diagnosis, management and prevention of poisonings (http://www.who.int/ipcs/poisons/centre/en/). Although their structures and functions are varying around the globe, such centres at least constitute an important information service. Service includes risk assessment and advice on therapeutic options in case of exposures to chemical agents, such as household products, industrial chemicals, pharmaceuticals and natural toxins. Promoting evidence-based and cost-effective treatment, as well as avoiding unnecessary or ineffective measures is among the main goals of all poisons centres.

The National Poisons Information Centre, Tox Info Suisse, provides 24-hour/7-days-a-week nationwide medical advice in cases of (suspected) poisoning to health professionals and the general public of Switzerland, which is currently at about 8.2 million people. Since its establishment in 1966, Tox Info Suisse has registered over one million poisoning-related phone calls. This potentially valuable source of nation-wide mycological information for the last five decades, however, has so far neither been described in a medical nor in an ecological context.

The main goal of this study is to introduce a novel dataset of spatiotemporal information on Swiss mushroom fruiting as recorded by the Poisons Centre inventory. A total of 12,126 mushroom-related phone calls to Tox Info Suisse between 1966 and 2014 were therefore digitized and homogenized. Intra- and interannual trends and signals in the Poisons Centre inventory are primarily described, but also initially compared with independent mycological evidence from the Swiss National Data Centre ‘SwissFungi’. Critical discussion associated with the newly developed Tox Info Suisse inventory emphasizes data incompleteness, imprecise species description, as well as contamination by cultivated and/or exotic mushrooms. The relevance of unravelling different drivers of the observed mushroom fruiting patterns is further stressed, and should be considered in any subsequent follow-up study.

## Materials and Methods

**Fig 1:**
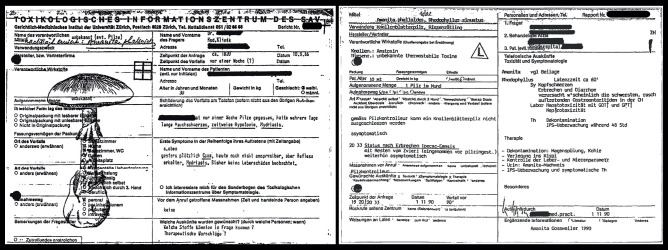
Two mushroom-related cases of Tox Info Suisse from the beginning of their recording period in 1966 (left) and later on in 1990 (right), which reveal slight changes in recording methodology over time.

Tox Info Suisse uses an electronic in-house database since 1994 allowing each phone call to be directly linked to the corresponding case record. Each individual case record contains the substance(s) involved, quantity (if known), route of exposure and demographic information, such as age, gender of the exposed person and (more or less precise) geographical origin of the call. Detailed clinical information on circumstances of exposure, symptoms and risk assessments is recorded in a systematic and standardized manner. Case records between 1966 and 1994 were solely written on paper files (see Fig 1 for two examples of different case files throughout time). Phone calls during the early period were only recorded if human or animal exposure was judged to be significant and symptoms were present or expected to occur. All paper files were later digitized and integrated into the newly developed, electronic Tox Info Suisse database as herein presented for the first time (S1 Table). Mushroom-related phone calls were labelled as precisely as possible with the relevant information provided by the caller. In case of significant risk, professional mushroom identification was advised. If available, the additional knowledge of the mushroom species was added to the case record.

A total of 12,126 cases of human mushroom exposure between 1966 and 2014 were extracted from the Tox Info Suisse database. This mushroom-related subset represents about 1% of the complete National Poisons Information Centre’s dataset, and includes the following items: Date and time of the phone call, ZIP code and/or canton from where the call originated, mushroom(s) by the scientific name or, if missing, the best available descriptive information, such as ‘mushrooms from the backyard’. All human mushroom exposures were included regardless of the origin of the mushroom (e.g. indigenous, exotic and/or cultivation). It is important to note that the extracted data do not include any clinical information (patient data, symptoms, treatment or outcome), and approval of the ethics committee was therefore not required. Due to slightly different manners of data collection throughout time, the inventory was adjusted in order to ensure temporal homogeneity. One case corresponds to one event, and one event is defined as one meal or one accident, regardless of the number of persons involved. Multiple case numbers related to the same person were eliminated, and only one record containing the most complete set of information was considered.

Although this study focuses on the introduction and description of a newly developed, ‘indirect’ source of mycological information (Tox Info Suisse), initial comparison steps were performed against independent evidence of intra- and interannual mushroom fruiting patterns as recorded by the Swiss National Data Centre ‘SwissFungi’ [39, 40]. SwissFungi is a community-driven and constantly growing project that is part of the national data centre for biodiversity initiated by the Federal Office for the Environment (FOEN). The main objective of SwissFungi is the update and presentation of species-specific fungal distribution maps for Switzerland, which subsequently serve as a base for the elaboration of the Red List of threatened species and inform authorities about presence of protection demanding species in each region. The success of SwissFungi strongly depends on the enthusiasm of volunteers and other generous data providers.

In this study, we mainly aimed at introducing and evaluating the potential of the Poisons Centre inventory for capturing myco-ecological information. Our preliminary analyses must therefore be understood as a proof of concept rather than a comprehensive ecological assessment. Being part of the Swiss National Data Centre for Biodiversity, SwissFungi currently comprises more than 540,000 fungal records, including species description as well as collection date and location. Focussing on macrofungi, most data originate from voluntary collaborators, scientific projects, museum collections or literature reports. As a main result, SwissFungi generates and updates fungal distribution maps across Switzerland, and also provides service for species conservation. SwissFungi data were herein aggregated on the taxonomic level of families following the ‘Index Fungorum’ [41].

## Results

**Fig 2:**
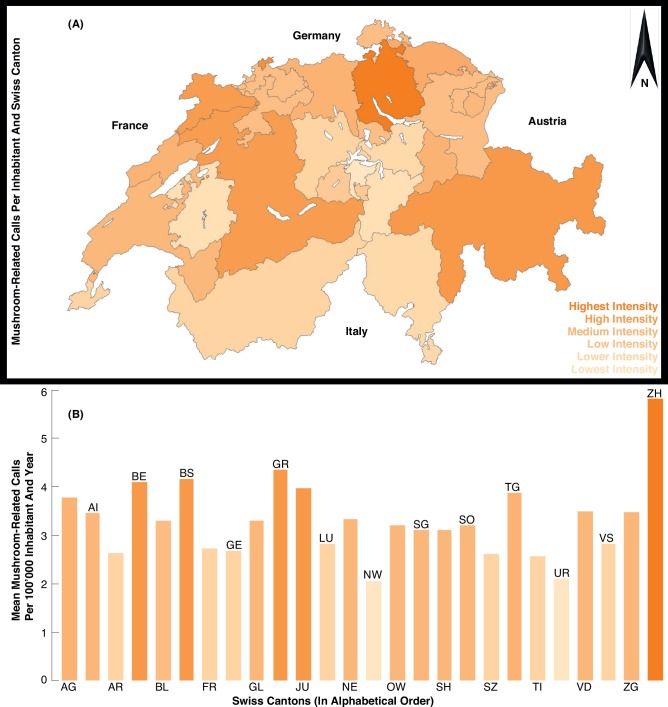
Spatial distribution of all 12,126 mushroom-related phone calls to Tox Info Suisse per 100,000 inhabitants at the cantonal-level.

The 12,126 mushroom-related calls to Tox Info Suisse are geographically well distributed across all 26 Swiss cantons (Fig 2A). The frequency of all calls relative to the population per canton is highest for Zurich, Grisons, Basle and Berne (>4.0 calls per 100,000 inhabitants), whereas the lowest numbers originate from Nidwalden and Uri (<2.5 calls). A maximum of 5.8 and a minimum of 2.1 is evident for Zurich and Nidwalden (Fig 2B), respectively.

**Fig 3:**
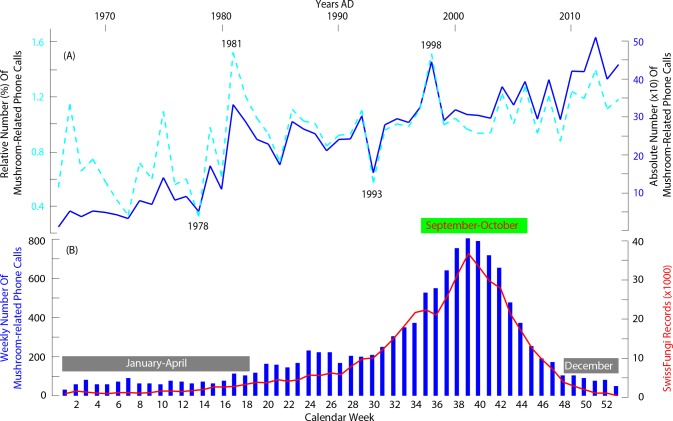
(**A**) Inter- and (**B**) intra-annual changes in all 12,126 mushroom-related calls to Tox Info Suisse (with the dashed light blue line in (**A**) referring to the relative number of calls in percentage), indicating national-wide trends and signals in mushroom fruiting patterns (i.e. productivity and phenology) over the last five decades. The weekly numbers in (**B**) equal the sum of all calls of a particular week from 1966–2014. Spring (March-May), summer (June-July) and autumn (September-November) is represented by the calendar weeks 09–22, 22–35 and 35–48, respectively.

The total and relative (%) numbers of mushroom-related calls steadily increased from 1966–2014 (Fig 3A), with additional year-to-year and decadal fluctuations being superimposed on the long-term trend. The absolute and relative time-series from Tox Info Suisse correlate (using Pearson’s correlation coefficient) highly significantly (*p* <0.001) with each other (*r* = 0.84). The strongest increase in the Poisons Centre inventory occurred in the 1980s and 1990s, whereas less variability is found over the last 15 years. Distinct depressions in the number of mushroom-related calls occurred in 1978 and 1993, whereas striking positive anomalies were recorded in 1981 and 1998. The observed mushroom fruiting trends and extremes are most reliable when expressed relative to the total number of Tox Info Suisse calls (see methods above). In this way, the overall long-term increase since 1966 is less pronounced (Fig 3A), but the annual anomalies are more distinct. Possible climatological and/or ecological explanations for both, the steady increase and the annual deviations in 1978, 1981, 1993 and 1998, however, will be subject to more environmental-oriented follow-up projects that must consider explicit ecological and climatological time-series for straightforward comparisons.

The weekly number of the Tox Info Suisse cases reveals a distinct seasonal pattern with a maximum at the transition of September and October (Fig 3B). This autumnal peak usually occurs in calendar week 39 (at the end of September/early October), when more than 800 (accumulated) calls per week have been so far registered (over the entire 1966–2014 recording). This autumnal fruiting peak is followed by a rapid declining to less than a total of 100 calls in December since 1966. An additional, though smaller intra-annual positive anomaly is found between week 24 and 26, which corresponds to the second half of June. This early summer peak was characterized by more than 200 weekly calls that were accumulated between 1966 and 2014. With less than 90 calls per week since recording start in the mid-1960s, the winter and spring months of January-April as well as December can be categorized as the classical mushroom off-season.

The intra-annual behaviour of all mushroom-related calls to Tox Info Suisse highly corresponds with that of the SwissFungi data (Fig 3B). Although the SwissFungi inventory is about 45 times larger, both datasets exhibit highest values in week 39 and also reveal nearly similar relative values from January-December. The observed phenological agreement between both datasets is most striking and clearly proofs the additional value of our newly generated Poisons Centre inventory for further ecological analyses (see also discussion for more details).

**Fig 4:**
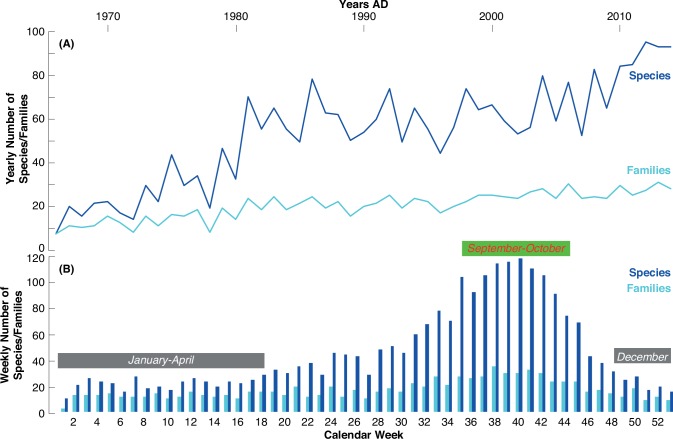
(**A**) Inter- and (**B**) intra-annual changes in the number of fungi species and families as recorded by Tox Info Suisse between 1966 and 2014. Spring (March-May), summer (June-July) and autumn (September-November) is represented by the calendar weeks 09–22, 22–35 and 35–48, respectively.

Around 20 different mushroom species were reported between 1966 and the early-1970s (Fig 4A). In the late-1970s a marked increase was observed and the yearly species numbers remained rather stable between 50 and 80 species until 2009, then climbing to 80–100 species in the last five years. Over the entire study period from 1966–2014, the number of mushroom species involved in human exposure has thus increased around 10 fold with seven species in 1966 and 93 in 2014. At the same time, the number of concerned mushroom families has doubled (Fig 4A). Seasonal changes in the numbers of species, as well as the number of families follow a very similar pattern compared to the overall mushroom-related Poisons Centre calls (Fig 4B). A maximum of fungal diversity is reached at the end of September and early-October with the accumulated occurrence of 40 mushroom families and more than 100 species per week (Fig 4B). In turn, the amount of species is highly significantly correlated with the number of mushroom-related Poisons Centre calls (r = 0.91).

**Fig 5:**
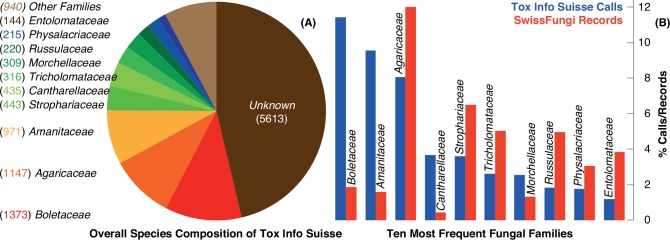
(**A**) The taxonomic affiliation of the 12,126 mushroom-related calls to Tox Info Suisse, and (**B**) the relative frequency of mushroom-related phone calls and SwissFungi records within the ten most frequently concerned fungal families of the Tox Info Suisse data source. A detailed overview on all species reported to Tox Info Suisse since 1966 is provided in the appendix **S1 Table.**

Species-specific information is available in 53.7% (n = 6,513) of all Poisons Centre cases (Fig 5A). The most frequently asked families are *Boletaceae*, *Agaricaceae* and *Amanitaceae* accounting for 11.3%, 9.5% and 8.0%, respectively. The category of ‘others’ (n = 940) comprises 50 families, among them *Cortinariaceae*, *Inocybaceae*, *Pleurotaceae* as well as *Omphalotaceae*, and accounts for 7.7%. Precise information on mushroom family or species was missing in 46.3% (n = 5613) of all calls. Most mushroom related calls to Tox Info Suisse relate to species in the family of *Boletaceae* and the most frequently concerned taxa is the King bolete (*Boletus edulis*, n = 741), followed by the most poisonous Death cap (*Amanita phalloides*, n = 555), the yellow Chanterelle (*Cantharellus cibarius*, n = 335), *Psilocybe* sp. (n = 245), as well as the Button mushroom or Portobello (*Agaricus bisporus*, n = 229). Comparison with SwissFungi shows a high coincidence in the taxonomic affiliation of the cases (Fig 5B). The top ten families, however, account for 46% of all mushroom-related calls to Tox Info Suisse, with the same ten families accumulating to 40% of all SwissFungi entries during the common period 1966–2014.

## Discussion and Conclusions

In light of the herein presented mycological achievements, it is important to note that any comparison between the two independent datasets at the family- and species-level is only partly informative, because the Tox Info Suisse inventory is certainly far away from representing a complete picture of the Swiss mushroom occurrence, and the overlap between both records is thus limited. Further analyses of the newly obtained, indirect evidence of fungal fruiting patterns should therefore focus on the general abundance level, instead of placing too much weight on individual taxa.

The mushroom-related calls to Tox Info Suisse are quite equally distributed over the 26 Swiss cantons. This spatial homogeneity of mushroom-related enquiries suggests that the Poisons Centre service is rather evenly considered throughout Switzerland. The distribution further underlines the potential of our dataset for nation-wide myco-ecological analyses and interpretations. The number of phone calls from canton Zurich, however, exceeded the number of calls from all other cantons. The same ratio is obtained when considering the entire Tox Info Suisse database of more than one million poisoning-related phone calls. Despite the geographically balanced distribution of mushroom-related calls to Tox Info Suisse, it is obvious that urban cantons (i.e. high population density and limited agricultural and forested areas) tend to exhibit more enquiries compared to rural cantons (i.e. low population density together with widespread agricultural and forested areas), which reflect an overall lower service demand. Service needs from Grisons in the eastern Swiss Alps, however, are an exception, as the second highest amount of phone calls per inhabitant originated from this canton. Parts of this canton are traditionally highly frequented by mushroom pickers, and Grisons is also a key destination for outdoor/hiking tourism. In summary, the newly developed Tox Info Suisse dataset reveals some spatial characteristics in mushroom-related phone calls, however, the extraction of geographically explicit myco-ecological information remains challenging.

There is a clear long-term positive trend in both, the absolute and relative numbers of mushroom-related calls to Tox Info Suisse since 1966, paralleled by a rising number of species and families. This increase in fungal productivity and diversity might be explained by a generally rising popularity in mushroom picking, or–most simply–by an increasing use of the Tox Info Suisse service due to a tendency towards decreasing mycological knowledge and experience of many of those collectors. Moreover, modern means of communication, such as online tools for mushroom identification might also affect use of the official mushroom control services. The recent generation of smart phone applications may additionally cause confusion and insecurity due to ambiguous online information and finally stimulate people to make use of the professional risk assessment delivered by the Poisons Centre. An increasing number of species might reflect more intensive investigations in species identification and an intensified collaboration with professional mycologists. The enhanced fungal diversity as captured by Tox Info Suisse is in accordance with results from a local-scale myco-ecological study [16, 42, 43]. In the fungus reserve ‘La Chanéaz’ a clear trend of increasing fruitbodies and species richness was observed over the last three decades. Nevertheless, the most significant boost in productivity and diversity occurred at the beginning of the 1990s [18]. In summary, the new Tox Info Suisse inventory provides a promising basis for more ecological- and environmental-oriented follow-up studies that aim at disentangling possible direct and indirect drivers of the observed year-to-year fluctuations and longer-term trends in the newly exposed Swiss mushroom fruiting patterns.

In addition to the observed temporal changes in fungal phenology and productivity, there is only little agreement between the taxa most frequently registered by Tox Info Suisse and those recorded by SwissFungi, which might be related to the fact that the two independent inventories are based on rather different concepts of data capturing. The SwissFungi project is taxonomically and spatially as complete as possible. In contrast, the Poisons Centre calls are biased towards intoxication, including species such as *Amanita phalloides* and *Psilocybe* species. Further bias of the Tox Info Suisse data may originate from the danger of misidentification, or simply by the quantities of consumption, including species such as *Boletus edulis*, *Cantharellus cibarius* and *Agaricus bisporus*, with the last mostly originating from cultivation. However, and most surprisingly, we found a high coincidence between the two datasets when comparing them at the family level. The percentage of calls within the top ten families of the Poisons Centre calls is nearly equal to that of the SwissFungi records (40% and 46%, respectively). Moreover, five of the top ten families in the Poisons Centre inventory also range in the top ten families of the SwissFungi archive.

Despite its advantages, our study is characterized by some uncertainty due to possible data incompleteness during the early recording period. The call registration process has changed during the study period from simple, hand-typed case cards, to a more sophisticated electronic dataset. There might also be a tendency towards underestimating the true number of edible species, because asymptomatic consumers of edible mushrooms generally do not call a poisons information centre for support. To some degree, there might even be erroneous species identifications inherent to the Tox Info Suisse dataset, as a professional mycologist did not systematically identify each case. Hence, there is the possibility that cultivated and/or exotic mushrooms were inadvertently included. This can happen, as specific mushroom species might be present on the market from cultivation (especially true for *Agaricus bisporus*), and from foraging simultaneously. In conclusion, caution must be advised when interpreting the newly developed Tox Info Suisse dataset.

Comparison between the mycological content of Tox Info Suisse with that of the SwissFungi project reveals a surprisingly high phenological agreement. Although being differently replicated, the intra-annual variability of the two datasets is nearly identical. A small exception is, however, expressed by a minor boost between week 24 and 26 in the Poisons Centre calls, which is not reflected by the SwissFungi data. This boost is driven by a specific group of fungal species fruiting primarily in that period and growing in parks and gardens, as well as on children’s playgrounds, commonly summarized as ‘lawn mushrooms’.

It is interesting to note in this regard, that data from poisons centres may even can help detecting new fungal species and associated toxicological risks. Examples include, for instance, plant material-contaminated edible mushrooms from Asia [44], as well as myco-toxicity of *Tricholoma equestre* [45]. Other cases of poisoning with chemicals are lung injuries due to inhalation of waterproofing sprays [46] or household surface treatment products [47], as well as 1,4-Butanediol-containing toys [48].

In conclusion, we can say that the mushroom-related calls to Tox Info Suisse mirror intra-annual phenology patterns of the real-word fungal fruiting compiled over Switzerland and the last five decades. In this regard, the newly developed, daily-resolved and spatially explicit Poisons Centre mycological dataset provides unique information on the dynamics of Swiss fungal productivity, phenology and diversity that occurred between 1966 and 2014. Despite stressing uncertainties related to citizen science-based data, this study emphasizes the potential of poisons information centres for providing historical myco-ecological insight at different spatiotemporal scales far beyond the main toxicological objectives of such services. Nevertheless, further assessments should be supplemented with high-resolution meteorological measurements as well as additional environmental indicators, such as phenological observations, as well as information on land-use/land-cover changes and pollution rates to help disentangling and better understanding the causes and consequences of high- to low-frequency variability in mushroom fruiting patterns, i.e. fungal phenology, productivity and diversity. Moreover, any ecological-oriented follow-up study should consider various caveats. Finally, we hope that the newly developed Tox Info Suisse dataset encourages the development of similar records in other countries, as well as the extraction and evaluation of other parameters from Poisons Centre inventories.

## Supporting Information

S1 Table This excel-file contains all data relevant to Figs 2–5. (XLSX)
